# From “Black Box” to a Real Description of Overall Mass Transport through Membrane and Boundary Layers

**DOI:** 10.3390/membranes9020018

**Published:** 2019-01-23

**Authors:** Endre Nagy, Márta Vitai

**Affiliations:** Chemical and Biochemical Processes Laboratory, Research Institute of Biomolecular and Chemical Engineering, University of Pannonia, Egyetem u. 10, H-8200 Veszprem, Hungary; vitai.marta@gmail.com

**Keywords:** “black box” model, two-layer transport, solution-diffusion model, diffusive plus convective flow, dense membrane, porous membrane, enhancement, polarization modulus

## Abstract

The “black box” model defines the enhancement, E the polarization modulus, $C^{*}/C^{o}$ and the intrinsic enhancement, $E_{o}$ without knowing the transport mechanism in the membrane. This study expresses the above-mentioned characteristic parameters, simultaneously taking into account the mass transport expressions developed for both the polarization and the membrane layers. Two membrane models are studied here, namely a solution-diffusion model characterizing solute transport through a dense membrane and a solution-diffusion plus convection model characterizing transport through a porous membrane due to transmembrane pressure difference. It is shown that the characteristic parameters of the “black box” model (*E,* $E_{o}$ or $C^{*}/C^{o}$) can be expressed as a function of the transport parameters and independently from each other using two-layer models. Thus, membrane performance could be predicted by means of the transport parameters. Several figures show how enhancement and the polarization modulus varied as a function of the membrane Peclet number and the solubility coefficient. Enhancement strongly increased up to its maximum value when *H* > 1, in the case of transport through a porous membrane, whereas its change remained before unity in the case of a dense membrane. When the value of *H* < 1, the value of *E* gradually decreased with increasing values of the membrane Peclet number.

## 1. Introduction

With rising numbers of various membrane separation processes, working with different transport principles, efforts for describing transport processes through the membrane layer have been substantially increased. The model description is generally based on different approaches, e.g., phenomenological ones (such as the so-called “black-box” model [1,2]), mechanistic models (which relate separation with structural membrane parameters, e.g., the solution-diffusion model (for nonporous, dense membrane layers)) [3,4,5], or non-equilibrium thermodynamics [6,7,8]. The “black box” model does not give any information on the transport process through a membrane separation unit. Its usage is especially advantageous when there is no real data on the structure of the membrane and accordingly its transport mechanism is not known [9,10,11]. For characterization of the separation process in this case, it uses additionally the measured outlet concentration value for description of the separation, which is considered to be a response of the membrane separation unit to the inlet parameter values. This “black box” model has often been used for characterization of the membrane separation process (e.g., reverse osmosis [8] or pervaporation [12,13]) in the starting time period of the membranes’ application for component separation. This model mostly characterizes membrane separation by the enhancement factor (this means the ratio of the outlet and inlet bulk solute concentrations), intrinsic enhancement (the ratio of the outlet concentration and that of the inlet membrane concentration), and the polarization modulus (the ratio of the inlet membrane concentration and inlet bulk concentration), applying the diffusion plus convection flows for the inlet polarization layer as well as the outlet convective flow [1,10,11,14].

The starting points of a simple mathematical model for a nonporous membrane layer, involving the terms of driving force, flux, and diffusion, are Fickian equations [4]. Accordingly, the flux is determined by the diffusion coefficient, the solubility coefficient, and also the driving force. This transport model is called the solution-diffusion transport model, and is often applied in, e.g., pervaporation and membrane gas separation processes [3,11]: It is also used for reverse osmosis in case of a nonporous active membrane layer [10]. In the case of mass transport through a porous membrane layer mostly involving both diffusive and convective flows [7], where often the convective one dominates the flow, the bulk flow is mostly negligible in transport through a nonporous membrane layer [5]. A solution-diffusion-imperfection model takes into account flow through small pores, depending on the hydraulic pressure difference, and was introduced by Sherwood et al. [15] for a reverse osmosis process and then was widely applied in the literature [16]. A typical example of the diffusion plus convection membrane process is nanofiltration as a pressure-driven membrane process, whose mass transport through a membrane was described by Bowen and Welfoot [17]. The bulk transport rate of the solvent phase through pores or a porous layer can be predicted by a Hagen-Poiseuille [11] (pp. 351–353), [18] (pp. 44–46) for straight line pores, or by Darcy equations [10,11,13,19], in which the solvent bulk velocity is expressed as linear dependence on the transmembrane pressure difference. 

The solute transfer rate is through a membrane in these kinds of membrane separation systems (e.g., nanofiltration, pressure-retarded osmosis, and forward osmosis), and in them the diffusion flux rate is comparable to values of convective transport rates and can be given as a sum of the diffusive and convective fluxes [11,17,20,21,22]. Diffusion plus convection mass transport can easily be extended to those accompanied by chemical or biochemical reactions [23,24,25,26]. When investigating membrane performance in the presence of convective velocity, the importance of the absence or presence of the sweeping phase on the permeation side of the membrane [11] (pp. 237–282) should be emphasized. Without the sweeping phase, the concentration gradient inside of the membrane layer can be generated only by a chemical or biochemical reaction. Namely, when the removal of the solute compound on the permeate side takes place through the convective velocity of the solvent phase, then the outlet concentration gradient, and also that inside of the membrane layer, is zero [11] (pp. 230–232). Only this latter type of membrane separation is discussed here.

The aim of this paper is to define characteristic parameters of the “black box” model, namely enhancement, intrinsic enhancement factors, and the polarization modulus, independently from each other, contrary to what is defined in the membrane “black box” models, as a function of the transport parameters, both of the polarization and the membrane layers. As membrane models, the solution-diffusion model and also the solution-diffusion plus convection models are applied, without the sweep phase on the permeate side. Such expressions are missing in the literature to our knowledge. Additionally, the overall mass transfer rate’s expressions are also expressed. 

## 2. Theory

In this section, we survey expressions of parameters used in the literature for applications of the “black box” model, namely the enhancement, the intrinsic enhancement, and the polarization modulus, and then the expressions developed by the authors for description of simultaneous transport through the polarization and membrane layers. The membrane transport expressions presented involve the solution-diffusion model and the solution-diffusion plus convection model with parameters of enhancement, intrinsic enhancement, a polarization modulus, and also overall mass transfer rate equations.

### 2.1. “Black Box” Model

**Figure 1:**
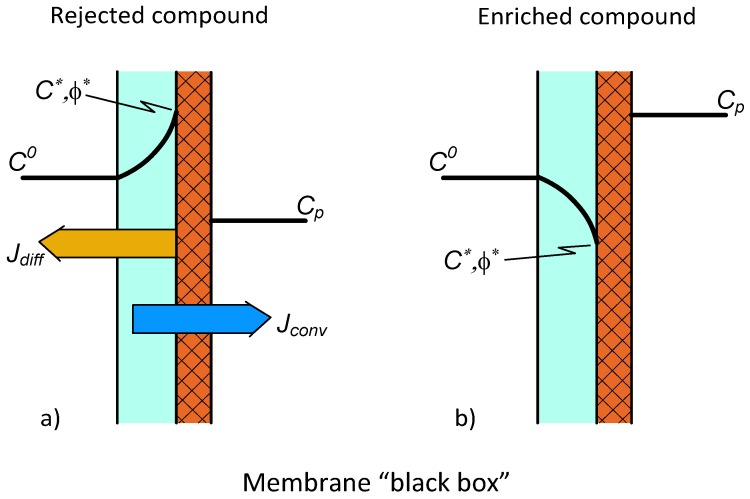
Concentration distribution in the polarization modulus assuming the membrane layer (right-side layer) to be “black box” with unknown mass transport properties. The diffusive and convective transports are in reverse (**a**) or concurrent directions (**b**).

The concentration distribution in the feed-side polarization layer is illustrated in Figure 1 for a rejected component (Figure 1a) and also for an enriched component (Figure 1b) [10,11]. These solute transport curves permit both the diffusive (directed to the bulk feed phase, Figure 1b, or directed to the permeate phase, Figure 1b) and convective flows (this latter flow transports the solute into the permeated phase [11] (p. 499)). The differential mass balance equation can be defined for both of these transports as [11] (p. 201)

(1) $$-D\frac{d^{2}C}{dy^{2}}+\upsilon \frac{dC}{dy}=0,$$

whereas the mass transfer rate for the polarization layer is [1,10,11]

(2) $$J^{o}=\upsilon C-D\frac{dC}{dy}.$$

The general solution of Equation (1) is (see for details Reference [11] (pp. 185–225))

(3) $$\phi =Te^{Pe}+S,$$

where

$$Pe=\frac{\upsilon \delta }{D}\equiv \frac{\upsilon }{k^{o}};\;k^{o}=\frac{D}{\delta }.$$

Parameters *T* and *S* can be obtained by means of suitable boundary conditions as (*Y = y*/δ),

(4) $$Y=\;0,\;\mathrm{then},\;\phi =\phi^{*},$$

(5) $$Y=\;1,\;\mathrm{then},\;\phi =\phi_{\delta }^{*}.$$

The concentration distribution then is obtained as

(6) $$\phi =\frac{\phi^{*}-\phi_{\delta }^{*}}{1-e^{Pe}}e^{PeY}+\frac{\phi_{\delta }^{*}-e^{Pe}\phi^{*}}{1-e^{Pe}}.$$

The mass transfer rate, using Equation (2), is then

(7) $$J^{o}=\beta^{o}\left(\phi^{*}-e^{-Pe}\phi_{\delta }^{*}\right),$$

where,

(8) $$\beta^{o}=\frac{\upsilon e^{Pe}}{1-e^{Pe}}\equiv k^{o}Pe\frac{1}{e^{-Pe}-1}.$$

In order to express the previously defined parameters in the “black box” model as enhancement, intrinsic enhancement, or a polarization modulus, the convective outlet flow rate (note if there is no feeding, inert phase (the so-called sweep phase) on the permeate side, then there is no diffusive flux into the permeate phase), and thus the solute permeate flux, should be given as

(9) $$J^{o}=\upsilon C_{p}=k_{L}^{o}Pe_{L}C_{p}.$$

This solute transfer rate then should be equal to the inlet solute rate defined by Equation (7). It is easy to get from the equality of Equations (7) and (9) the following, regularly applied, literature expression [1]:

(10) $$\frac{C^{*}-C_{p}}{C^{o}-C_{p}}=e^{Pe_{L}}.$$

Let us express the generally used definition for enrichment, *E*, ($E=C_{p}/C^{o}$), and intrinsic enrichment, $E_{o}$ ($E_{o}=C_{p}/C^{*}$), as well as the polarization modulus ($=C^{*}/C^{o}$). Values of *E* can vary from unity up to very big values (in the case of enriched solute) or from unity down to zero (in the case of rejected solute). Similarly, the polarization modulus can be higher or lower than unity (see Figure 1 for this). By application of Equation (10) and the values of intrinsic enhancement, the enhancement factor can be expressed as a function of the intrinsic enhancement as [10]

(11) $$\frac{C_{p}}{C^{o}}=E=\frac{E_{o}e^{Pe_{L}}}{1+\left(e^{Pe_{L}}-1\right)E_{o}}.$$

From this, one can predict the value of the polarization modulus, namely

(12) $$\frac{C^{*}}{C^{o}}=\frac{E}{E_{o}}=\frac{e^{Pe_{L}}}{1+E_{o}\left(e^{Pe_{L}}-1\right)}.$$

On the other hand, the intrinsic enhancement, which cannot separately be determined, can be given as a function of the value of *E* as

(13) $$E_{o}=\frac{E}{E\left(1-e^{Pe_{L}}\right)+e^{Pe_{L}}}.$$

Equations (11)–(13) unambiguously show that neither the enhancement factor and the intrinsic enhancement factor nor the polarization modulus can independently be expressed (through application of the “black box” model) independently from each other as a function of measurable transport parameters without knowing the transport properties within the permselective membrane layer.

### 2.2. Solute Transport Using the Solution-Diffusion Model for the Membrane Layer 

**Figure 2:**
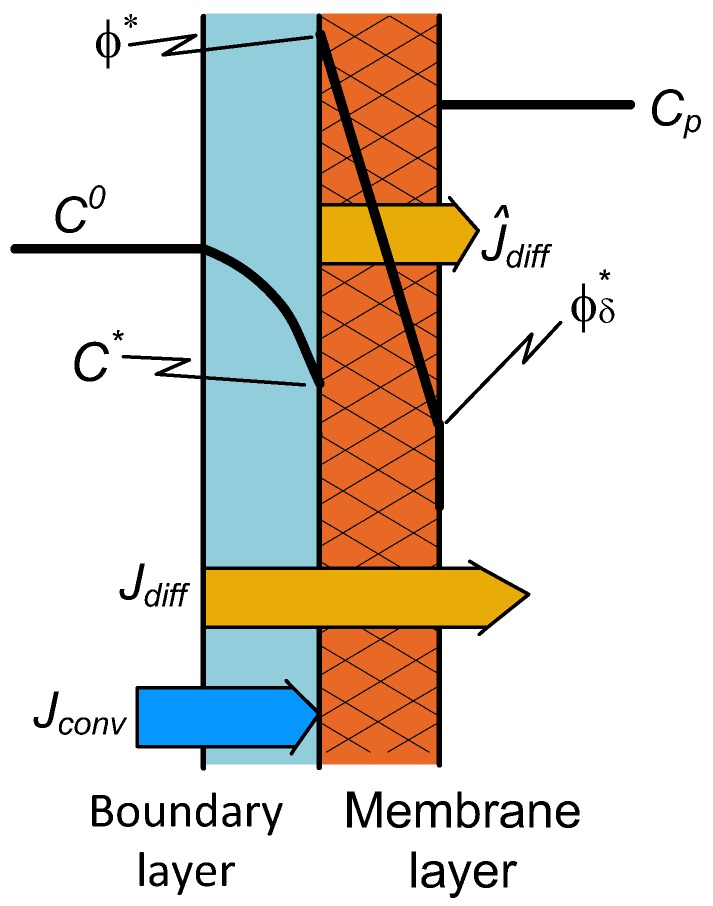
Schematic illustration of the concentration distribution for the case of a dense membrane with an “enriched” solute component.

Solute transport through the dense membrane is mostly described by the solution-diffusion model, as it is the case, e.g., during pervaporation [27], membrane gas separation [28,29], or pressure-retarded osmosis [30]. In the first two cases, there is no sweep phase on the permeate side: A vacuum in the outlet phase continuously moves the permeated compound(s) away from the outlet membrane surface. Due to this, the concentration of the permeated compound is close to zero on the outlet membrane surface. Thus, its value can be much less than that of an equilibrium concentration with a condensed permeate phase. The concentration distribution in the two transport layers is illustrated in Figure 2. Accordingly, a simple solution-diffusion mass transfer process takes place through the dense (selective) membrane layer. Thus, the solute flux for the membrane layer is

(14) $$J^{o}=k^{o}\left(\phi^{*}-\phi_{\delta }^{*}\right)\equiv k^{o}H\left(C^{*}-C_{p}\right).$$

The two fluxes, namely those given for the boundary layer and for the membrane layer, given by Equations (7) and (14), respectively, are equal to each other, and thus the overall mass transfer rate can be expressed using this equality. Accordingly, it is ($\phi^{*}=HC^{*}$ and $HC_{p}=\phi_{\delta }^{*}$) [11],

(15) $$J_{ov}^{o}=\beta_{ov}^{o}\left(C^{o}-e^{-Pe_{L}}C_{p}\right),$$

where

(16) $$\beta_{ov}^{o}=\frac{k_{L}^{o}Pe_{L}}{1+e^{-Pe_{L}}\left(N-1\right)},$$

with

(17) $$N=\frac{k_{L}^{o}Pe_{L}}{Hk^{o}}\equiv \frac{\upsilon }{Hk^{o}}\equiv \frac{\upsilon \delta }{HD}.$$

Note that it is assumed in Equation (15) that the outlet membrane concentration and the neighboring fluid concentration are in equilibrium. Parameter *N* expresses the ratio of the convective velocity in the boundary layer to the product of the solubility coefficient and the membrane diffusive mass transfer coefficient. Now let us express the enhancement, the intrinsic enhancement, and the polarization modulus independently from each other, contrary to what is given in the “black box” model, by using the mass transport parameters of the polarization and the dense membrane layers.

For prediction of the concentration distribution inside the transport layers, the interface concentration of the layers should be determined. Applying Equations (7) and (15), it can be written

(18) $$-\beta_{L}^{o}\left(C^{o}-e^{-Pe_{L}}C^{*}\right)=\beta_{ov}^{o}\left(C^{o}-e^{-Pe_{L}}C_{p}\right).$$

Expressing $C^{*}$ from Equation (18), one gets the concentration polarization modulus as [11] (pp. 210–213)

(19) $$\frac{C^{*}}{C^{o}}=e^{Pe_{L}}+\frac{\beta_{ov}^{o}}{\beta_{L}^{o}}\left(\frac{C_{p}}{C^{o}}-e^{Pe_{L}}\right).$$

After transformation of Equation (19), one gets ($N=k_{L}^{o}Pe_{L}/Hk^{o}$),

(20) $$\frac{C^{*}}{C^{o}}=\frac{\left(1-e^{-Pe_{L}}\right)\frac{C_{p}}{C^{o}}+N}{1+e^{-Pe_{L}}\left(N-1\right)}.$$

The enhancement factor can easily be expressed by using the equality of the outlet and inlet streams,

(21) $$k_{L}^{o}Pe_{L}C_{p}=-\beta_{ov}^{o}\left(C^{o}-e^{-Pe_{L}}C_{p}\right)\equiv \frac{k^{o}Pe_{L}}{1+e^{-Pe_{L}}\left(N-1\right)}\left(C^{o}-e^{-Pe_{L}}C_{p}\right).$$

Thus, this is

(22) $$\frac{C_{p}}{C^{o}}=\frac{\beta_{ov}^{o}}{k_{L}^{o}Pe_{L}+\beta_{ov}^{o}e^{-Pe_{L}}},$$

or after rearrangement,

(23) $$E=\frac{C_{p}}{C^{o}}=\frac{1}{1+Ne^{-Pe_{L}}}.$$

Replacing the value of *E* from Equation (23) into Equation (20), one gets the polarization modulus, taking into account the solute transfer rate in the membrane layer, as

(24) $$\frac{C^{*}}{C^{o}}=\frac{\left(1-e^{-Pe_{L}}\right)\frac{1}{1+Ne^{-Pe_{L}}}+N}{1+e^{-Pe_{L}}\left(N-1\right)}.$$

Taking into account that $C^{*}/C^{o}=E/E_{o}$ and Equations (20) and (23), the intrinsic enhancement is

(25) $$E_{o}=\frac{1+e^{-Pe_{L}}\left(N-1\right)}{1-e^{-Pe_{L}}+N\left(1+e^{-Pe_{L}}N\right)}.$$

The value of the intrinsic enhancement can be obtained without taking into account the transport rate inside the membrane. Thus, its value can be obtained by equality of the outlet flow (Equation (7)) and inlet flow, expressed by the boundary layer resistance (Equation (9)) only. Thus, one gets for the polarization modulus [11] (pp. 213–214)

(26) $$\frac{C^{*}}{C^{o}}=\frac{1+N}{1+Ne^{-Pe_{L}}},$$

and one gets for the intrinsic enhancement

(27) $$E_{o}=\frac{1}{1-e^{-Pe_{L}}}\left(\frac{C_{o}}{C^{*}}-e^{-Pe_{L}}\right).$$

Replacing the reciprocal value of the polarization modulus into Equation (27), the value of $E_{o}$ is

(28) $$E_{o}=\frac{1}{1+N}.$$

It is obvious that the values of the polarization modulus given by Equations (25) and (28) are equal to each other in a special case only, because Equation (28) has a restriction caused by the membrane transport resistance. It is easy to get that the equality of these two equations is fulfilled only when [11]

(29) $$N=\frac{e^{Pe_{L}}\left(1-E\right)}{E}.$$

This equation follows from Equation (23) as well.

### 2.3. Two-Layer Transport with Diffusion Plus Convection for Both the Transport Layers

**Figure 3:**
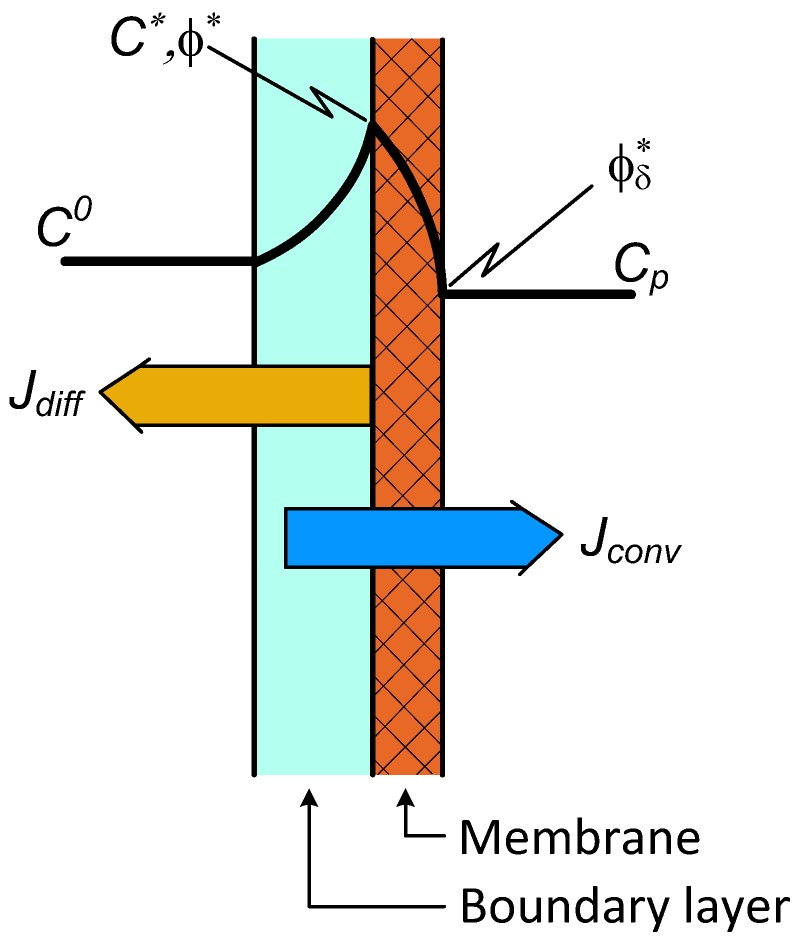
Illustration of the two-layer concentration distribution with convective transport in the membrane layer as well.

The concentration distributions and nominations are illustrated in Figure 3. The mass transfer rate for the boundary layer is expressed by Equation (7), while that for the membrane layer is

(30) $$J^{o}=\upsilon S\equiv \beta^{o}\left(\phi^{*}-e^{-Pe}\phi_{\delta }^{*}\right),$$

with

(31) $$\beta^{o}=k^{o}Pe\frac{1}{e^{-Pe}-1}.$$

The overall mass transfer rate, obtained through equality of these two transfer rates, namely Equations (7) and (30), is

(32) $$J_{ov}^{o}=\beta_{ov}^{o}\left(C_{L}^{o}-e^{-\left(Pe+Pe_{L}\right)}C_{p}\right),$$

with

(33) $$\frac{1}{\beta_{ov}^{o}}=\frac{e^{-Pe_{L}}}{H\beta^{o}}+\frac{1}{\beta_{L}^{o}}.$$

Another form of the overall mass transfer coefficient is

(34) $$\beta_{ov}^{o}=\frac{k_{L}^{o}Pe_{L}Pe}{Pe\left(e^{-Pe_{L}}-1\right)+\left(e^{-Pe}-1\right)Ne^{-Pe_{L}}}\;.$$

Through equality of the transport rate for the polarization layer and the overall solute transfer rate, the polarization modulus can be expressed as

(35) $$\beta_{L}^{o}\left(C^{o}-e^{-Pe_{L}}C^{*}\right)=\beta_{ov}^{o}\left(C^{o}-e^{-\left(Pe+Pe_{L}\right)}C_{p}\right).$$

Thus, the polarization modulus is then obtained from Equation (35) as [11] (pp. 204–207)

(36) $$\frac{C^{*}}{C^{o}}=\frac{Pe\left(e^{-Pe_{L}}-1\right)e^{-Pe}\frac{C_{p}}{C_{o}}+\left(e^{-Pe}-1\right)N}{Pe\left(e^{-Pe_{L}}-1\right)+\left(e^{-Pe}-1\right)Ne^{-Pe_{L}}}.$$

The enhancement factor can be expressed (similarly to Equation (21)), applying its equality here with Equation (32), as the overall mass transfer rate:

(37) $$E\equiv \frac{C_{p}}{C^{o}}=\frac{Pe}{{}^{Pee^{-\left(Pe+Pe_{L}\right)}-\langle Pe\left(e^{-Pe_{L}}-1\right)+\left(e^{-Pe}-1\right)Ne^{-Pe_{L}}\rangle }}.$$

Knowing the value of $C^{*}/C^{o}$ (Equation (36)) and the enhancement factor, the intrinsic enhancement factor is given as

(38) $$E_{o}=\frac{E\left\{Pe\left(e^{-Pe_{L}}-1\right)+Ne^{-Pe_{L}}\left(e^{-Pe}-1\right)\right\}}{N\left(e^{-Pe}-1\right)+Ee^{-Pe}\left(e^{-Pe_{L}}-1\right)Pe}.$$

Replacing the value of the enhancement (Equation (37)) into Equation (38), the intrinsic enhancement can also be given as a function of the mass transport parameters, namely *N* ($N=k_{L}^{o}Pe_{L}/Hk^{o}$), *Pe*, and $Pe_{L}$.

Let us look at, in the following sections, how the defined characteristic parameters *E*, $E_{o}\;C^{*}/C^{o}$ change as a function of the transport parameters.

## 3. Results and Discussion

The main point of this study is to show how the most popular, characteristic parameters of membrane separations (enhancement, intrinsic enhancement, and the polarization modulus) can be predicted, taking into account not only the transport parameters of the feed boundary (polarization) layer (diffusive and convective flows as well as the solubility coefficient between the fluid and the membrane phases), but also the transport parameters of the membrane layer. Nowadays, the expressions of mass transport through both the dense and porous membrane layers are well elaborated [9,10,11,12]. How the characteristic parameters of the “black box” model can be predicted independently from each other as a function of the transport parameters of both the transport layers has not been discussed in the literature yet to the authors’ knowledge. Thus, the discussion of how the enhancement, intrinsic enhancement factors, and polarization modulus vary as a function of the simultaneous effect of two-layer solute transports can be useful and instructive, and it can significantly contribute to a better understanding of the real meaning of membrane separation properties. It can also help in the prediction of the performance of planned membrane processes. 

The transport mechanisms in membrane layers are very complex, but are widely discussed processes. They strongly depend on both the membrane and the solute/solvent properties. Here, two main types of transport, namely diffusive and diffusive plus convective transport, were considered depending on the membrane structure properties. The solution-diffusion transport model was used for transport through dense membranes, and the solution-diffusion plus convection model was used for describing transport through a porous membrane layer. All transport parameters, namely diffusion coefficients in the fluid and solid membrane layers, convective velocity, and the solubility coefficient, were assumed to be constant. How these parameters depend on operating conditions (e.g., transmembrane pressure) or on fluid/membrane properties (e.g., physical or chemical properties, membrane structure) were not topics of this study. 

It is worth noting that parameters defined by the “black box” (Equations (11)–(13)) are correct, real values. Their only insufficiency is that the parameters are not expressed independently from each other, and thus their absolute values cannot be given by expressions obtained by the “black box” model. In order to do this, the transport process in the membrane layer should also be involved, which enables users to express these parameters as a function of transport parameters only, independently from each other.

### 3.1. Transport with Dense Membranes

**Figure 4:**
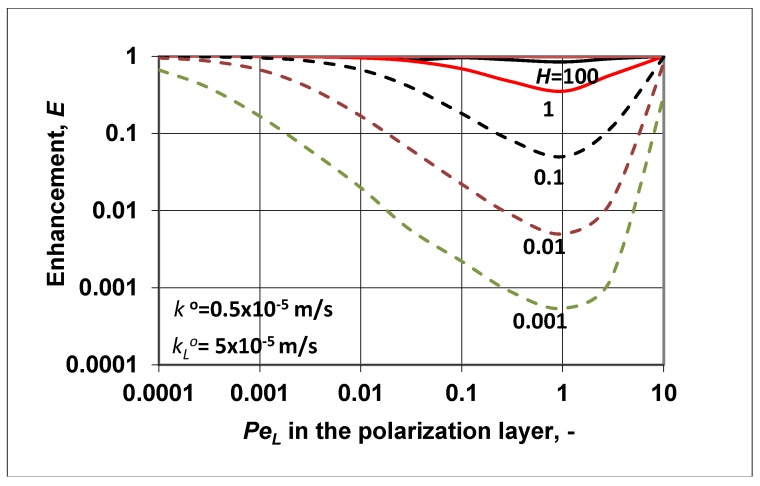
Enhancement as a function of $Pe_{L}$ at different values of the membrane solubility coefficient. It was assumed that to get the enhancement, the outlet convective flow was equal to the overall solute transfer rate (Equation (21)); $k_{L}^{o}$ = 5 × 10^−5^ m/s; $k^{o}=k_{L}^{o}/10$.

The application of such membranes, mostly asymmetric membranes with very thin, dense, selective layers, is typical in the case of, e.g., pervaporation, membrane gas separation, or pressure driven separation processes. In this study, focusing on the characteristic parameters *E*, $E_{o}$ and polarization modulus ($C^{*}/C^{o}=E/E_{o}$), the simultaneous effect of the dense membrane layer and feed side polarization layer were taken into account, defined by Equations (23), (25), and (24), respectively. Both parameters, namely *E* and $E_{o}$ were expressed as a function of the *N* parameter ($N=\upsilon /Hk^{o}$; see Equation (17)) and of the boundary layer’s convective velocity, or more exactly, of $Pe_{L}$ Variables *N* and $Pe_{L}$ involve the diffusive mass transfer coefficient of the transport layers, the convective bulk velocity in the polarization layer, and the solubility coefficient, *H* (H=ϕ/C), as well. Let us show the change in the enhancement factor (Figure 4) as a function of the fluid phase Peclet number at different values of *H* and, consequently, at different values of *N*. 

**Figure 5:**
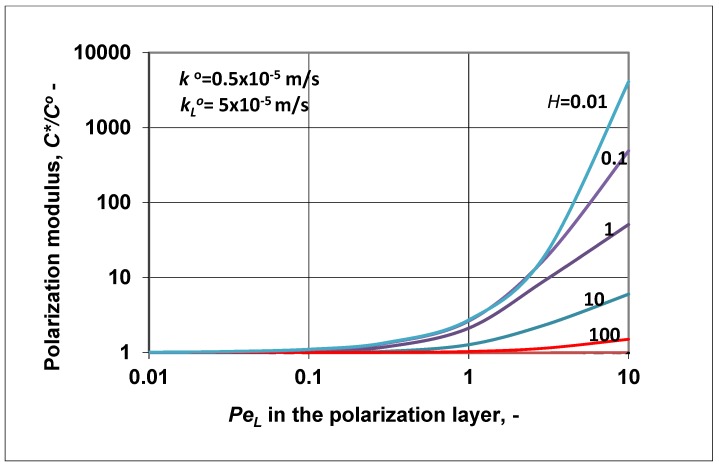
Polarization modulus as a function of the Peclet number of the polarization layer, at different values of the solubility, calculated by Equation (24); $k_{L}^{o}$ = 5 × 10^−5^ m/s; $k^{o}=0.1k_{L}^{o}$.

According to Equation (23), it is obvious that the value of the enhancement factor could not be higher than unity. This means that the value of the outlet solute concentration was not higher than its inlet one. This is perhaps surprising, because according to Figure 1b, the value of $C_{p}$ should be higher than the inlet value in the feed phase at high values (higher than unity) of solubility. On the other hand, for the case of $C_{p}/C^{o}>1$, the polarization modulus should be lower than unity. As we can see later in Figure 5, this condition was also not fulfilled. How, then, can real enhancement be achieved by a nonporous, dense membrane, where there are no pores with enough size, and thus bulk fluid transport cannot be created? This not-desired phenomenon should have been caused by our assumption that the outlet membrane concentration was in equilibrium with the outlet fluid concentration, i.e., $\phi_{\delta }^{*}=HC_{p}$. To reach real enrichment of the outlet solute component, the outlet membrane concentration should be less than its equilibrium concentration, i.e., $\phi_{\delta }^{*}=HC_{p}$. This assumption was fulfilled during pervaporation through the application of vacuum pressure on the permeate side (this case is discussed in detail in Reference [11] (pp. 217–219)), and also during membrane gas separation using lower pressure on the permeate side than on the inlet side.

On the other hand, let us briefly discuss the curves (broken lines in Figure 4) when *H* < 1. For this case, the shape of the enhancement curves was as expected. With a decrease in the solubility coefficient, the outlet solute concentration also decreased, increasing the separation efficiency of the membrane process. The curves had minimum values as a function of the convective velocity, which did not induce component separation. The minimum of the curves fell to approximately$Pe_{L}=1$. Further increasing the convective velocity gradually decreased the separation performance, and the enhancement values gradually approached unity.

Let us show how the value of the polarization modulus changed as a function of $Pe_{L}$ (Figure 5), using the same values for the transport parameters as used in Figure 4. As was expected, the value of the inlet membrane concentration increased as a function of the bulk phase velocity, in harmony with the enhancement data plotted in Figure 4. High values (higher than unity) of the polarization modulus at *H* > 1 might mean that the resulting convective transport rate (subtracting the reverse diffusive flow from the convective one) was still high, comparing it to the diffusive one in the membrane layer. This resulted in an increase of the $C^{*}$ value to equalize inlet flow rate with that occurring in the membrane layer. Obviously, the higher value of $C^{*}/C^{o}$ lowered to unity with the increase of the membrane diffusive mass transfer coefficient (not shown here), but its value could not decrease below unity, according to Equation (24).

Note that by placing the values of enhancement or intrinsic enhancement (obtained by Equations (23) or (25)) into those obtained by the “black box” model (given in Equations (11) and (13), respectively) (e.g., the *E* value obtained by Equation (23) is placed into Equation (13) for calculation of the intrinsic enhancement factor), one gets back exactly the same values as were obtained by Equations (23) or (24). Let us look at an example with the following parameters: $k_{L}^{o}=5\times 10^{-5}$m/s; $k^{o}=k_{L}^{o}/10$; *H* = 0.1; $Pe_{L}=0.01$, and thus *N* = 1. The calculated values of the presented models are *E* = 0.5025 (Equation (23)), $E_{o}=0.5000$ (Equation (25)). Placing the value of *E* into Equation (13) (obtained by the “black box” model), the value of intrinsic enhancement $E_{o}$ is equal to 0.4999. Placing $E_{o}=0.5000$ into Equation (11), we can get *E* = 0.5025, which is equal to that obtained by the presented model. This proves that both model expressions are correct if one of them is accepted to describe correctly the real transport process. Accordingly, knowing the transport parameters of the layers (mass transfer coefficients, solubility, convection velocity), the above model can be used to predict the separation performance of, e.g., pervaporation or membrane gas separation, whose processes use dense membrane layer for separation.

Comparing the results plotted in the above figures to those given by Baker [10] (pp. 177–179), there might be a stated qualitative difference between their information contents. The presented model gives direct data on the effect of the two-layer transport process, while the literature ones can give connection values between the enhancement and the intrinsic enhancement or polarization model only. 

The methodology presented here can also be used in the case of variable membrane diffusion coefficients or solubility coefficients. In this case, the transport rate through the membrane should also be contained by their dependency on, e.g., the concentration and temperature. This problem can be an object of another study.

### 3.2. Transport with Convection in a Porous Membrane Layer

This section is important for pressure-driven membrane separation processes (e.g., nanofiltration, ultrafiltration) using porous membranes. Separation of the transport components by these so-called filtration processes depends mainly on particle and pore sizes. The bulk convective flow can mostly be predicted by Darcy’s law [10,11,12]. The transport equations given for these membrane processes are listed in Section 2.3. The characteristic parameters, namely enhancement, intrinsic enhancement, and the polarization modulus, are defined by Equations (37), (38), and (36), respectively. These three equations tend toward those ones defined by the solution-diffusion models (Equation (23)–(25)) when the convective velocity is eliminated, i.e., when *Pe* → 0. 

**Figure 6:**
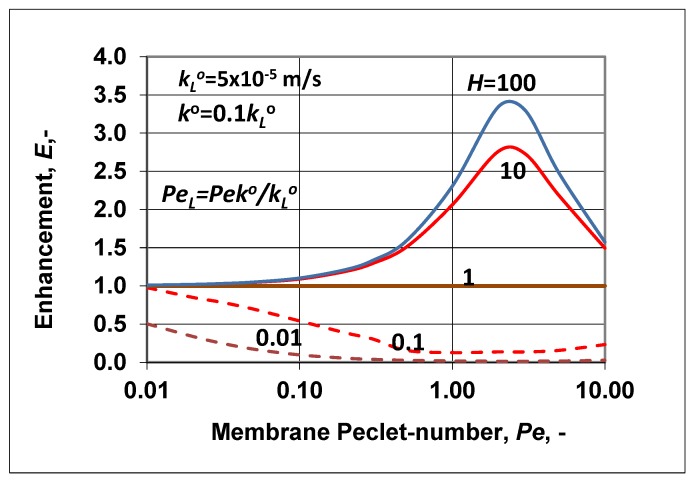
Enhancement as a function of the membrane Peclet number, *Pe*, at different values of the solubility coefficient ($Pe_{L}=Pek^{o}/k_{L}^{o}$; $k_{L}^{o}$ = 5 × 10^−5^ m/s; $k^{o}=0.1k_{L}^{o}$).

Let us look at some predicted results obtained for values of enhancement and the polarization modulus. The intrinsic enhancement is not an independent parameter, namely $E_{o}=E/\left(C^{*}/C^{o}\right)$, and thus its value is not plotted here. Figure 6 shows the change of enhancement, predicted by Equation (35), as a function of the membrane Peclet number. The solubility coefficient (according to the value of *N*) was chosen to be higher (continuous lines) or lower (broken lines) than unity. The change of the outlet concentration ($E=C_{p}/C^{o}$), as expected, strongly depended on the value of the solubility coefficient, *H*. At values of *H* > 1, the *E* value gradually increased to a value of the membrane convective velocity. With further increases of the membrane convective velocity, the value of *E* started to gradually decrease down to unity. This means that the membrane separation property gradually lost its separation efficiency due to the nonselective convective velocity. With increasing values of *H*, the outlet concentration also increased depending on the value of the membrane Peclet number, *Pe*. At a value of *H* = 1, there was now separation of the solute, and the outlet solute concentration was equal to the inlet one. When the solubility value was less then unity, this was the solubility range of solute rejection (see Figure 1a), and the outlet concentration decreased as a function of the membrane convective velocity, increasing the membrane separation efficiency. When *Pe* > 1, the *E* value started to increase slowly, after its minimum. This means that the separation efficiency decreased in this *Pe* range (*Pe* > 1). 

**Figure 7:**
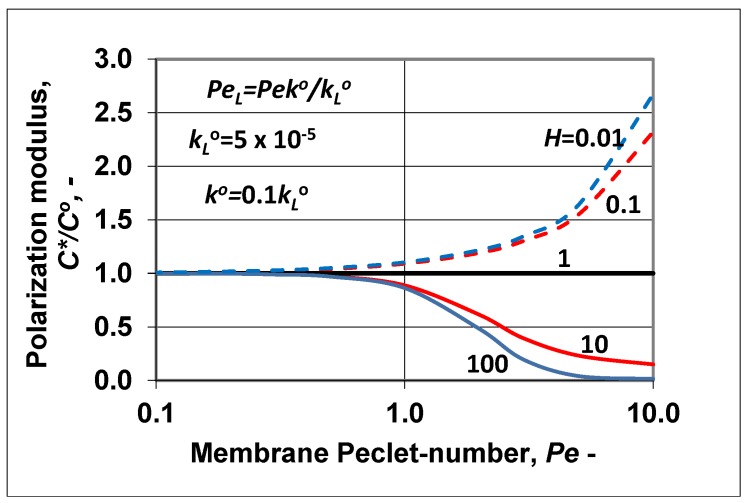
Polarization modulus as a function of the membrane Peclet number, calculated by Equation (36), at different values of the solubility coefficient, *H*. Values of the mass transfer coefficient and of the $Pe_{L}$ of the polarization layer are the same as in Figure 6, namely ($Pe_{L}=Pek^{o}/k_{L}^{o}$; $k_{L}^{o}$ = 5 × 10^−5^ m/s; $k^{o}=0.1k_{L}^{o}$).

The change of the polarization modulus ($=C^{*}/C^{o}$) is plotted in Figure 7 as a function of the membrane Peclet number. The values of the parameters $k^{o}$, $k_{L}^{o}$, *H* were chosen to be the same as they were in Figure 6. The polarization modulus was calculated by Equation (36). Obviously, trends of its changes were in harmony with the results plotted in Figure 6 and also with the curves plotted in Figure 1a (*H* < 1; in Figure 7, broken lines) and Figure 1b (*H* > 1; here, continuous line). Values of the polarization modulus continuously lowered as a function of the membrane convective velocity in the membrane Peclet regime investigated. At values of *H* ≥ 100, its value was close to zero. Obviously, the value of *N* also changed as a function of *Pe*, because the absolute value of the convective velocity in the polarization layer was the same as that in the membrane layer due to the assumed expression of $Pe_{L}=Pek^{o}/k_{L}^{o}$. Obviously, the slopes of the curves strongly depended on the ratio of the diffusive mass transfer coefficients. Increasing the membrane mass transfer coefficient, $k^{o}$ at a constant value of $k_{L}^{o}$ its value increased and the values of the polarization modulus were lower (in the enrichment solute regime) or higher (in the case of rejected solute) (not shown here). These two figures, namely Figure 6 and Figure 7, clearly prove that the methodology shown for prediction of the characteristic membrane parameters defined in the “black box” model can be correctly used for two-layer transport processes with convective flow in both the polarization and porous membrane layers as well.

**Figure 8:**
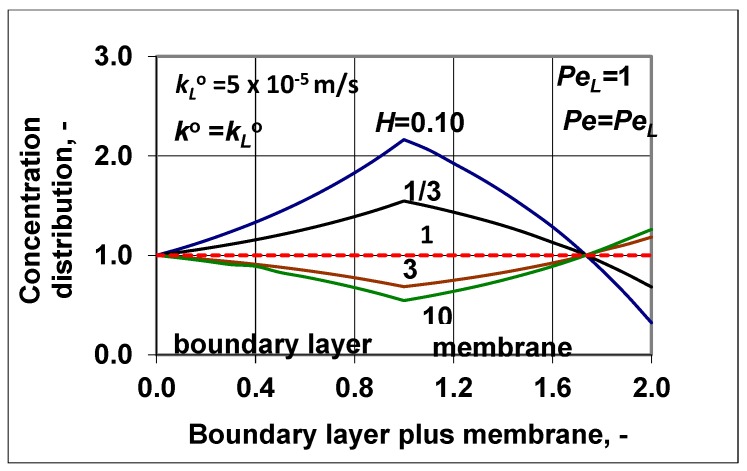
Concentration distribution in the feed side polarization layer and the membrane layer at a given value of the convective velocity and the diffusive mass transfer coefficient as well as at different values of the solubility coefficient; ($Pe_{L}=Pe=1$; $k_{L}^{o}$ = 5 × 10^−5^ m/s; $k^{o}=k_{L}^{o}$).

It might also be interesting for readers to see how the concentration distribution changed in the two transport layers (Figure 8), applying values obtained for the feed-side membrane interface concentration (values taken from the polarization modulus obtained) and for the outlet ones (using enhancement data). The values of the polarization modulus obtained were 0.545, 0.635, 1.546, and 2.163, and the values of the enhancement obtained were 1.26, 1.083, 0.682, and 0.323 at *H* = 10, 3, 1/3, and 0.1, respectively. At *H* = 1, both parameters got values of unity (continuous red line in Figure 8). It can be said that the curves’ curvatures were typical. The curves were concave and convex for cases of *H* < 1 and *H* > 1, respectively. On the other hand, the role of convective velocity was crucially important. Its value could strongly increase the separation efficiency with increases or decreases in the values of *E*, i.e., with increases or decreases in the outlet solute concentration. Thus, by checking or adjusting the transmembrane hydraulic or osmotic pressure difference and other operating conditions, an optimal or desired separation efficiency can be reached, whose advance could be very essential in separation performance, as is well illustrated in Figure 6. With knowledge of the transport parameters (the diffusive mass transfer coefficients, the convective velocity, and solubility), the desired separation properties can be predicted by the expressions presented in this study. 

The methodology presented can also be applied to the separation of colloids, oil droplets in water, macromolecules, etc., by applying pressure-driven separation processes (e.g., nanofiltration, ultrafiltration).

## 4. Conclusions

The solute transport process developed by the “black box” model, which was published several decades ago, was extended for two-layer transport, taking into account the transport “mechanisms” of the membrane layers as well. Transport expressions for membranes have already been given in the literature depending on the membrane structure and properties. It was shown in this study that completing the boundary layer’s transport expression with that given for the membrane layer, the more important characteristic parameters (enhancement, intrinsic enhancement, and polarization modulus) can be expressed independently from each other as a function of the diffusive mass transfer coefficient, convective velocity (if it exists in the membrane at all), and the solubility coefficient between the fluid and membrane phases (if it exists). This extension was done and discussed in this study using solution-diffusion (i.e., pervaporation, membrane gas separation) and solution-diffusion plus convection transport in a porous membrane layer (i.e., nanofiltration, ultrafiltration). It was shown that the solution-diffusion membrane model did not give higher than unity enhancement values at *H* > 1, applying the usually used outlet boundary condition, whereas it gave expected results when *H* < 1. The values of enhancement and the polarization modulus, using the diffusion-convection model for transport in a porous membrane, changed as was expected. The independent expressions of enhancement and the polarization modulus enable users to predict separation performance as a function of two-layer transport parameters (diffusion coefficients, convective velocity, solubility coefficient), which can significantly contribute toward being chosen as operating conditions that can provide desired separation efficiency.

## Nomenclature

*C*	concentration in the fluid phase, kg/m^3^
*D*	diffusion coefficient, m^2^/s
*E*	enhancement, -
$E_{o}$	intrinsic enhancement, -
*H*	solubility coefficient, kg/kg
*J* ^o^	mass transfer rate, kg/m^2^s
$k^{o}$	membrane diffusive mass transfer coefficient, m/s
$k_{L}^{o}$	diffusion coefficient in the feed fluid phase, m/s
*Pe*	Peclet number (Equation (3))
*y*	local coordinate, m
β	mass transfer coefficient in presence of convective velocity, m/s
ϕ	solute concentration in the membrane, kg/kg
**Superscript:**	
*	interface
o	inlet
**Subscript:**	
*L*	fluid phase
*p*	outlet
